# Transnasal targeted delivery of therapeutics in central nervous system diseases: a narrative review

**DOI:** 10.3389/fnins.2023.1137096

**Published:** 2023-05-19

**Authors:** Seoyeon Won, Jeongyeon An, Hwayoung Song, Subin Im, Geunho You, Seungho Lee, Kyo-in Koo, Chang Ho Hwang

**Affiliations:** ^1^College of Medicine, Chungnam National University, Daejeon, Republic of Korea; ^2^Major of Biomedical Engineering, Department of Electrical, Electronic, and Computer Engineering, University of Ulsan, Ulsan, Republic of Korea; ^3^Department of Physical and Rehabilitation Medicine, Chungnam National University Hospital, College of Medicine, Chungnam National University, Daejeon, Republic of Korea

**Keywords:** therapeutics, central nervous system, wounds and injuries, targeted delivery, nanoparticles, exosomes, magnetics

## Abstract

Currently, neurointervention, surgery, medication, and central nervous system (CNS) stimulation are the main treatments used in CNS diseases. These approaches are used to overcome the blood brain barrier (BBB), but they have limitations that necessitate the development of targeted delivery methods. Thus, recent research has focused on spatiotemporally direct and indirect targeted delivery methods because they decrease the effect on nontarget cells, thus minimizing side effects and increasing the patient’s quality of life. Methods that enable therapeutics to be directly passed through the BBB to facilitate delivery to target cells include the use of nanomedicine (nanoparticles and extracellular vesicles), and magnetic field-mediated delivery. Nanoparticles are divided into organic, inorganic types depending on their outer shell composition. Extracellular vesicles consist of apoptotic bodies, microvesicles, and exosomes. Magnetic field-mediated delivery methods include magnetic field-mediated passive/actively-assisted navigation, magnetotactic bacteria, magnetic resonance navigation, and magnetic nanobots—in developmental chronological order of when they were developed. Indirect methods increase the BBB permeability, allowing therapeutics to reach the CNS, and include chemical delivery and mechanical delivery (focused ultrasound and LASER therapy). Chemical methods (chemical permeation enhancers) include mannitol, a prevalent BBB permeabilizer, and other chemicals—bradykinin and 1-O-pentylglycerol—to resolve the limitations of mannitol. Focused ultrasound is in either high intensity or low intensity. LASER therapies includes three types: laser interstitial therapy, photodynamic therapy, and photobiomodulation therapy. The combination of direct and indirect methods is not as common as their individual use but represents an area for further research in the field. This review aims to analyze the advantages and disadvantages of these methods, describe the combined use of direct and indirect deliveries, and provide the future prospects of each targeted delivery method. We conclude that the most promising method is the nose-to-CNS delivery of hybrid nanomedicine, multiple combination of organic, inorganic nanoparticles and exosomes, via magnetic resonance navigation following preconditioning treatment with photobiomodulation therapy or focused ultrasound in low intensity as a strategy for differentiating this review from others on targeted CNS delivery; however, additional studies are needed to demonstrate the application of this approach in more complex *in vivo* pathways.

## 1. Introduction

The main central nervous system (CNS) treatments used today, including neurointervention, surgery, medication, and CNS stimulation (Daroff et al., 2016), are often invasive, extending the patient’s recovery and rehabilitation time. While the injection of neurotropic drugs such as chondroitinase (Kasinathan et al., 2016), anti-nogo (Zörner and Schwab, 2010), and Rho antagonists (Bertrand et al., 2005) has been considered a replacement for these other treatments in *in vitro/in vivo* CNS injury models, this method affects not only diseased tissues but also nonrelated healthy tissues (Perez-Herrero and Fernandez-Medarde, 2015). Therefore, CNS therapeutics have recently focused on targeted spatiotemporal therapy to rapidly induce the accumulation of high-concentration therapeutics in damaged areas, thereby making the treatment more intelligent, safer, more effective, and more efficient than the aforementioned methods.

**Figure 1:**
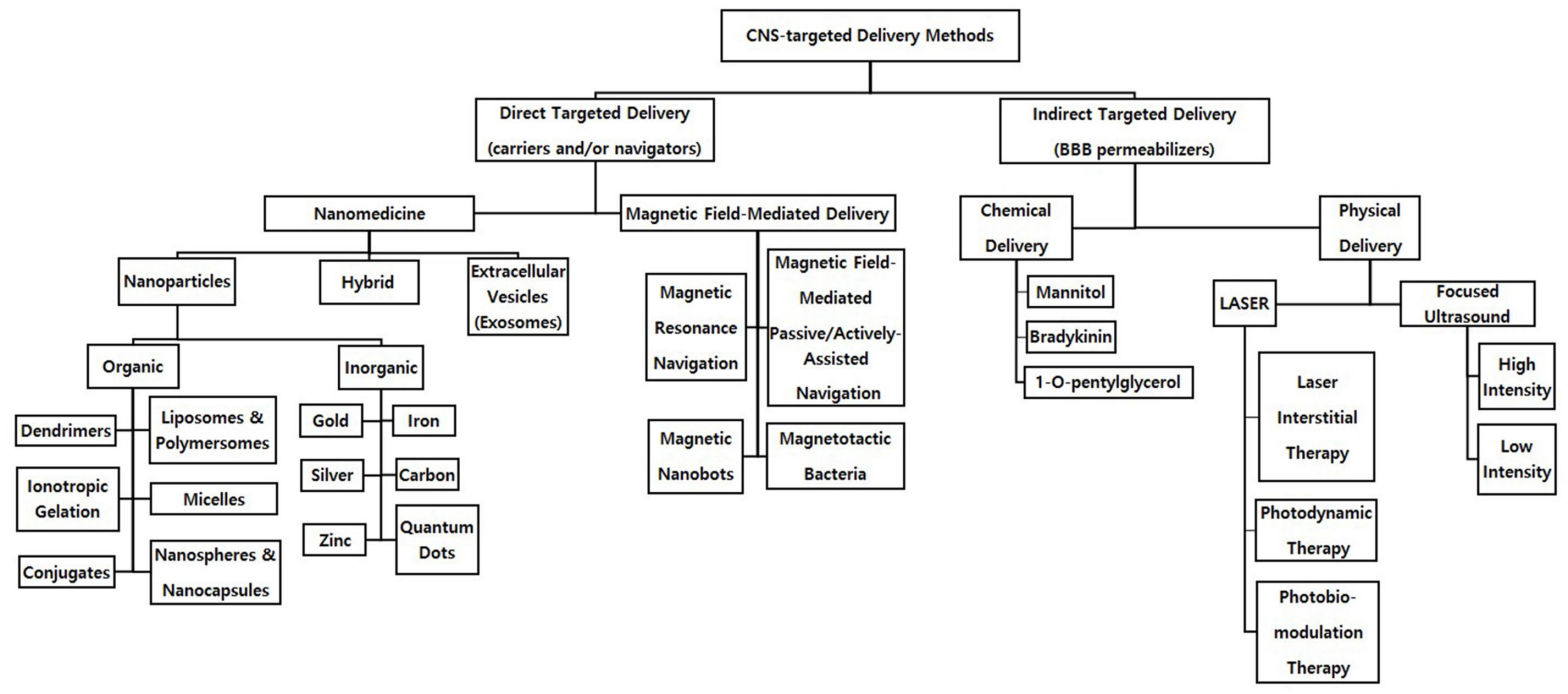
Direct and indirect targeted delivery methods to the central nervous system.

To achieve targeted delivery into the CNS, it is essential for therapeutic agents to pass directly through the blood brain barrier (BBB; Chacko et al., 2013). The BBB is a semipermeable layer of endothelial cells between the circulatory system and the CNS. While the BBB functions to protect the CNS from toxic substances and regulate ions, molecules, and cells for proper neuronal function (Daneman and Prat, 2015), its high selectivity, in which enzymatic and physical barriers are involved, also prevents therapeutics from reaching the CNS (Tewabe et al., 2021). Therefore, direct delivery methods such as nanoparticles (NPs; Dong, 2018; Tewabe et al., 2021), extracellular vesicles (EVs), and magnetic-field mediated delivery (Yu et al., 2018), and indirect delivery methods such as chemical and physical delivery methods are being developed to pass through the BBB and directly affect the CNS: direct delivery methods that allow therapeutics to pass through the BBB as a vehicle or a navigator versus indirect delivery methods that increase the permeability of the BBB as a permeabilizer (Figure 1).

While the direct and indirect methods have been mainly used separately, the authors of this review seek to shed light upon cases where the two methods are used together to create a synergistic effect and introduce the limitations and/or remaining unknowns in the field in regard to their combination. The direct and indirect CNS-targeted delivery strategies introduced in this article have demonstrated promise in *in vitro* and in animal studies as the next-generation platform for the treatment of CNS diseases ranging from Alzheimer’s disease to brain tumors. Therefore, this review aims to describe various direct and indirect CNS-targeted delivery methods and how they relate to one another to stimulate more interest and research in CNS-targeted delivery by defining the mechanism of each delivery method, discussing their advantages and disadvantages, and analyzing future perspectives for neuroscientists who may aim to understand or even use some of the ideas discussed here in the context of their own work. In addition, although numerous types of therapeutic agent administration, such as subcutaneous, intramuscular, intravascular, transintestinal, transperitoneal, and intrathecal ones, have been tried, all of these routes are currently being evaluated in pioneering studies and thus demand further investigation prior to discussion about whether these approaches have any validity on the CNS-targeted delivery of therapeutics. Just one type of administration, nose-to-CNS route, has been discovered as a promising tool in animal and human models, especially for neurological disease treatment (Mittal et al., 2014). Hence, it should be in mind that the current review is full of examples depicting nose-to-CNS delivery, using viral vectors for delivery, etc.

## 2. Direct targeted delivery

In direct CNS-targeted delivery, therapeutics may be preserved by methods such as encapsulation to permeabilize through the BBB safely, arrive in the CNS, and take effect. Among variety of direct methods in progress, nanomedicine and magnetic field-mediated deliveries are the most commonly used methods in a field of CNS disease treatment. Nanomedicine allows sustainable selective delivery to the target tissue (Dong, 2018; Tewabe et al., 2021), and magnetic field-mediated deliveries incorporate an external or internal magnetic field to directly navigate and accumulate magnetic therapeutics to the targeted area, resulting in a higher controllability than nanomedicine (Yu et al., 2018). Both nanomedicine and magnetic field-mediated deliveries could be seen as effective, promising delivery platforms for CNS diseases ranging from acute stroke to neurodegenerative disease to brain tumors, which have been most affected by the advancements made in direct delivery methods (Tiebosch et al., 2012; Tietze et al., 2013; Tentillier et al., 2016).

### 2.1. Nanomedicine

There are three types of namomedicine: NPs, EVs, and hybrid. NP structures, including 1D nanowires, 2D nanosheets, and 3D structures (Huang et al., 2020), such as carbon nanotubes (CNTs), liposomes, micelles, dendrimers, nanocapsules/nanospheres, and polymeric NPs (Perez-Herrero and Fernandez-Medarde, 2015). This review focuses on 3D structures since they allow for easier encapsulation and additional protection of therapeutics. The current review divides NPs into organic and inorganic type. Such classification of NPs focuses on their biocompatibility and stability depending on the consistency of the drug-containing shell. Also, this review introduces EVs, especially exosomes, which are the most up-to-date nanomedicine. Recent studies have focused on hybrids, as they minimize the disadvantages and maximize the advantages of organic, inorganic NPs, and EVs by combining the two or more (Ferreira Soares et al., 2020). Additionally, nanomedicine may be magnetized and incorporated in magnetic field-mediated delivery.

### 2.2. Organic nanoparticles

Organic NPs consist of organic materials, including lipids and aqueous molecules (Xie et al., 2019), allowing for high biocompatibility. Usually, NPs (liposomes, dendrimers, etc.), are prepared with functional moieties such as polyethylene glycol (PEG). For example, organic NPs made of lipids break down into lactic and glycolic acids after the loaded therapeutics are released into the target area, which can then be used up in the metabolic cycle (Vanden-Hehir et al., 2019). Additionally, organic NPs such as dendrimers, which are polymeric molecules with numerous branch structures enabling their surface to be easily modified, exhibit high bioavailability (Palmerston Mendes et al., 2017). When combined with organic NPs, the unstable hydrophobic drugs showed improved dissolution and stability (Pentek et al., 2017). For instance, the 100% dissolution time of resveratrol, a compound often known as an antioxidant, was found to be approximately 4.5 h when tested alone, while it took approximately 0.5 h in dendrimer-drug complexes (Chauhan, 2018). Additionally, 90% of resveratrol remained in dendrimer-resveratrol complexes compared to 10% for pure resveratrol, which indicates that dendrimers can mitigate stability issues (Pentek et al., 2017). Furthermore, organic NPs have been designed to mitigate the issue of crossing the BBB so that such liposomes and micelles have been reported to protect their vehicles from degradation and transport their loaded therapeutics across the BBB (Spuch and Navarro, 2011; Zou et al., 2017). For another example, the CD163 receptor-targeted liposomes incorporating dexamethasone showed a 3-fold higher delivery rate in the brain than sham group in 6-hydroxydopamine Parkinson’s disease rat models (Tentillier et al., 2016). The solid lipid NPs encapsulating acetylcholinesterase reactivators with the help of multiple emulsion technique presented a 9-fold higher delivery rate than acetylcholinesterase reactivator only group 45 min after injection in organophosphate-induced brain injury rat models (Pashirova et al., 2017).

Another notable advantage of organic NPs is their allowance for active cellular targeting; ligand tagging techniques allow the attachment of ligands, such as antibodies, peptides, aptamers, and folate molecules to the NP surface. The ligands make the organic NPs bind to target cell receptors, leading to efficiently selective delivery with high therapeutic efficacy (Mullen and Banaszak Holl, 2011); the L-carnitine-conjugated poly lactic-co-glycolic acid NPs containing paclitaxel showed a 10-fold higher accumulation in the brain than paclitaxel only injection 2 h after administration in brain tumor mouse models (Kou et al., 2018). In a review article in 2021, apolipoprotein E-conjugated polymeric NPs were emphasized regarding their merits such as the improved transport across the BBB, accumulation in the brain, and inhibition of the accumulation of neurodegenerative disease-related factors (Hartl et al., 2021). Similar to the protein derivative conjugation, glucose-coated micelles piled up in the mouse brains 56-fold higher than micelles without glucose conjugation (Anraku et al., 2017).

Despite exhibiting high biocompatibility, high bioavailability, high penetrability of the BBB, and high targetability, the use of organic NPs still faces several obstacles. One major problem is the difficulty of large-scale production. For example, liposome production currently involves numerous steps, increasing the complexity of the entire manufacturing process and making commercialization difficult (Roces et al., 2020). Moreover, light, temperature, and metal ions may trigger chemical changes in the NPs such as lipid oxidation, leading to permeability changes when these NPs could be exposed to such changes (e.g., during transportation, in storage, in the body after administration; Guimarães et al., 2021). Although promising results—the variety of polymer coatings has been developed to prevent the removal of NPs by mononuclear phagocyte systems, which almost recognize the administered NPs as a foreign body and make those defending systems activated, before they would arrive in the targeted CNS (Owens and Peppas, 2006) and coating NPs with PEG is the most effective manner to prohibit nonspecific protein adherence and subsequent phagocytosis—have been reported (Vonarbourg et al., 2006), potential problem with organic NPs such as liposomes is that they can still accumulate in the liver and spleen; this accumulation triggers phagocyte uptake by mononuclear phagocyte systems, dominantly by Kupffer cells in the liver, and 99% of the administered NPs can be eliminated (Campbell et al., 2018). Additionally, it is another issue that microglia facilitate NP clearance in the CNS, capturing even PEGylated NPs (Gu et al., 2020).

Many promising studies are in progress to overcome these limitations (Guimarães et al., 2021). For example, microfluidics used in liposome manufacturing can simplify and scale up production while enhancing drug encapsulation using “bottom-up” approaches, which have no needs of additional size reduction process (Forbes et al., 2019). Moreover, controlling cholesterol, phosphatidic acid, and PEG content in liposomes has been shown to improve stability (Szabová et al., 2021). However, studies on these areas are limited, these may be described as possible future directions for the development of organic NPs. Despite the efforts to mitigate the limitations of organic NPs, recent studies have focused on hybrid NPs and indirect methods (Shah et al., 2020). But still, the biocompatible, bioavailable, BBB-penetrable characteristics of organic NPs are noteworthy (Table 1).

**Table 1:** Types of nanoparticles.

Type	Advantage/Disadvantage		Authors	Subject	Result
Organic	Advantage	High biocompatibility/low immunogenicity	Vanden-Hehir et al. (2019)	Organic NPs made of lipids	Break down after delivering the drug to the target area, and are used up in the metabolism cycle
		Dissolution and stability improvements	Chauhan (2018)	Dendrimers combined with organic NPs	100% dissolution time took about 0.5 h but 4.5 h when tested alone
			Pentek et al. (2017)	Resveratrol in dendrimer-drug complexes	90% remained compared to 10% in pure resveratrol
	Disadvantage	Difficulty of large-scale production	Roces et al. (2020)	Producing organic NPs involves numerous steps	Increases the complexity of the entire manufacturing process and serves as an obstacle to commercialization
		Instability (chemical degradations by metal ions)	Guimarães et al. (2021)	Lipid oxidation	Lead to permeability changes
		Early elimination	Campbell et al. (2018)	Organic liposomes	The phagocyte system in the liver can take up to 99% of the administrated NPs
Inorganic	Advantages	High drug-carrying capacity	Kostarelos et al. (2005); Perez-Herrero and Fernandez-Medarde (2015); Wu et al. (2009)	HCPT covalently attached to the surface of a multi-walled carbon nanotube	Showed a sufficient loading efficiency of approximately 16%
			Cheng et al. (2019)	MSNs with a large internal pore volume	Resulting in a vast carrying capacity of 100 wt% for anticancer drugs
		Surface Modifiability/Effective Targeting	Dhar et al. (2008)	CNTs with the folate compound	Allowed for specific delivery of the therapeutics to FR(+) cancer cells
			Cheng et al. (2013)	Au-SMCC-DOX nanoconjugates	Showed effective drug accumulation in hepG2-R cancer cells
	Disadvantages	Toxicity problem	Ali-Boucetta et al. (2011)	AuNPs accumulated for over 1 month in the spleen and liver of rats	Changed the expression of genes, squalene epoxidase showed fold change of 2.9 ± 0.7, *p* < 0.05
			Ali-Boucetta et al. (2011)	Cells incubated with polymer-coated multi-walled nanotube: F127	F127 (concentration of 125 μg/ml) for 24 h and 48 h showed 60 and 40% of cell viability
Hybrid	Advantages	Tunable size	Bose et al. (2020)	Antibiotic-loaded LPHNPs engineered with CA and ZA lipids	NA was shown to have a diameter of 226 ± 9.6 nm, CA LPHNPs were 203 ± 6.6 nm and ZA LPHNPs were 191 ± 5.4 nm in diameter,size of hybrid NPs are tunable
		Surface charge		CA or ZA LPHNPs introduced into the LPHNP formulation in the same study	Charge reduction of −29 ± 2.1 mV occured
		High drug loading yield	Bose et al. (2020)	Comparing the %EE and vancomycin %EE of BNPs and CA or ZA lipid layered LPHNPs	The %EE of both CA and ZA LPHNPs is greater than that of bare organic NPs
		Sustained drug release	Bose et al. (2020)	Cumulative antibiotic release from doxycycline-encapsulated BNPs and vancomycin-encapsulated BNPs	The antibiotic release rates of doxycycline BNPs at 12, 24, and 48 h were each 65, 69, and 75%
		High stability	Yang J. et al. (2021)	*In vivo* gene delivery study; bare pCas9/MGMT degraded within 3 min of incubation with DNase I, and the pCas9/MGMT plasmids in LPHNs-cRGD	Remained intact throughout varying incubation durations with DNAse I		
		Biocompatibility	Khan et al. (2020)	CCK-9 assay results of LPHN-cRGD	Demonstrated cell viability of >80% after exposure to varying concentrations of NPs
		Efficient selective delivery and higher therapeutic efficacy by tagging lignads	Anraku et al. (2017)	Glucose-administered micelles for the treatment of Alzheimer’s	Showed a 56-times higher accumulation rate in the CNS than micelles without ligands
			Hartl et al. (2021)	Conjugation of apolipoprotein E(Apo E) with NPs	Improved transport across the BBB, accumulation in the brain, and inhibition of the accumulation of neurodegenerative disease-related factors
	Disadvantages	Unbalanced ratio of the components, less controlled antigen release, lipid layer not fully stabled, and unprotected hybrid structure integrity during long-term storage	Hu et al. (2010)		
		Harmful tissue deposition in the kidneys, reticuloendothelial system, and microvasculature, unintended activation of host immune response, and damage to target cells	Howard et al. (2014)	ALAs	

NPs, nanoparticles; HCPT, hydroxycamptothecin; MSNs, mesoporous silica nanoparticles; CNTs, carbon nanotubes; FR, folate-receptor; AuNPs, gold nanoparticles; LPHNPs, lipid-polymer hybrid nanoparticles; CA, cationic; ZA, zwitterionic; %EE, encapsulation efficiencies; BNPs, bare nanoparticles; CNS, central nerve system; and BBB, blood–brain barrier.

### 2.3. Inorganic nanoparticles

Inorganic NPs made of materials such as gold, iron oxide, and carbon have a high surface area to volume ratio (Yao et al., 2020), which enables a high drug-carrying capacity. For example, CNTs have a diameter of 0.4–3.0 nm and a length of 20.0–1000.0 nm, resulting in a high carrying capacity; when the antitumor agent 10-hydroxycamptothecin, which has been investigated for the treatment of brain glioma, was covalently attached to the surface of a multiwalled CNT, it showed a sufficient loading efficiency of approximately 16% (Kostarelos et al., 2005; Wu et al., 2009; Perez-Herrero and Fernandez-Medarde, 2015). Additionally, mesoporous silica NPs have a large inner pore amount, making them advantageous drug-carrying agents (Yao et al., 2020) with a carrying capacity of 100 wt% for anticancer drugs in mouse models (Cheng et al., 2019). The surface modifiability of inorganic NPs enables effective targeting; for example, attaching a folate-containing compound to CNTs enabled specific delivery of therapeutics to folate-receptor + cancer cells (Dhar et al., 2008; Perez-Herrero and Fernandez-Medarde, 2015). Gold NPs, Au-SMCC linker-doxorubicin nanoconjugates, also displayed surface modifiability made by connecting doxorubicin to the surface of gold NPs and showed effective drug accumulation in HepG2-R cancer cells (Cheng et al., 2013). Like aforementioned findings, modified gold NPs on strategies for CNS-targeted delivery have gained considerable attention as the relevance to the context of CNS disease treatment including brain tumors. In addition to the high drug-carrying capability, inorganic NPs such as silver, gold, or quantum dots can cross the BBB with their own mechanisms; gold NPs increased the BBB permeability by disrupting the endothelial tight junctions, in which Na, K, and/or Ca ion channels are be involved, and reached their the peak concentration in the cerebrospinal fluid (CSF) 3-fold higher than that in the blood 6 h after transperitoneal injection in rats, and quantum dots conjugated with fluorescein hydrochloride, which uses glucose transporters to penetrate the BBB, accumulated in the central canal and in the cervical spinal cord, especially in the gray matter, following transcardic and intravenous injection in zebrafishes and rats, respectively (Sela et al., 2015; Seven et al., 2021). Also, multi-walled CNTs, which use energy-dependent transcytosis, completely crossed the BBB and remained persistently in the brain even 24 h following intravascular injection in rats while notable decrement occurred in the peripheral capillaries (Kafa et al., 2015).

Despite the advantages of inorganic NPs, they exhibit immunotoxicity, genotoxicity, or specific tissue toxicities such as neurotoxicity, leading to inflammation, carcinogenesis, fibrosis, cardiovascular diseases, etc. (Mohammadpour et al., 2019). For example, after a month of accumulation in the spleen, gold NPs have been shown to downregulate the expression of genes relevant to wound healing, responses to external stimuli, and defense responses (Balasubramanian et al., 2010). In the liver, genes relevant to lipid metabolism or the cell cycle process were upregulated (e.g., squalene epoxidase expression showed a fold change of 2.9; Balasubramanian et al., 2010). Additionally, cells incubated with polymer-coated multiwalled nanotube F127 for 24 and 48 h showed 60 and 40% cell viability, respectively, indicating cytotoxicity (Ali-Boucetta et al., 2011).

However, inorganic NPs can be controlled by regulating size, morphology, dosage, and chemical factors to regulate their toxicity (Madani et al., 2011). During the generation of NPs, doping, a technique in which impurities are added to the NPs for chemical/physical improvements, may be used to reduce cytotoxicity (George et al., 2010). In doping methods, three types are commonly used; first, elemental doping is conducted at atomic level, and divided into metal doping using such as cerium, cobalt, copper, iron, lanthanum, manganese, potassium, silver, and zinc as dopants, among which manganese is widely used, and nonmetal doping using such as boron, carbon, fluorine, nitrogen, phosphorus and sulfur, among which nitrogen is the mostly applied dopant. Second, it can be performed at molecular level (molecular doping), in which active molecules are encapsulated into vehicles such as metal–organic frameworks, polymers, quantum dots, and silica, among which silica is the mostly used matrix. Third, codoping combines the benefits of codoped elements, through which metal combination, such as iron and sulfur, nitrogen and sulfur, copper and manganese, as well as combination of metal and nonmetal dopants have been achieved (Wang et al., 2022). However, such relationship between the matched dopants and NP matrix is not clearly known so that further studies will be required to establish a guideline for reliable manufacture of doped NPs.

Additionally, iron oxide NPs can be degraded into Fe ions in lysosomes of cells or under other acidic conditions, reducing the potential long-term toxicity (Yu et al., 2012; Mitchell et al., 2021). In a long-term (approximately 13 months) study regarding the safety of ferumoxytol, an FDA-approved iron oxide NP, in the treatment of iron-deficient anemia patients with chronic kidney disease on hemodialysis, no patients experienced intravenous ferumoxytol replacement treatment-related serious adverse events (Macdougall et al., 2019), indicating the relevance of the safety of iron NPs in the context of long-term administration. If toxicity issues can be resolved, inorganic NPs could have a high potential for use in effective direct delivery methods in the treatment of CNS disease due to their ability to easily cross the BBB and their efficient drug loading capacity (Table 1).

### 2.4. Extracellular vesicles/exosomes

While NPs are in common synthetic drug delivery vehicles (Sun et al., 2022), EVs are innate, nano-sized, phospholipid bilayer-enclosed vesicles (Elsharkasy et al., 2020) that can be, in theory, released by all kinds of cells (Rufino-Ramos et al., 2017) through exocytosis for intercellular communication. Recently, EVs have gained attention as a delivery tool of therapeutics owing to their groundbreaking preclinical success in brain-targeted delivery. Though the degree may vary depending on the type, EVs hold notable advantages for CNS-targeted delivery of therapeutics because of their noninvasiveness, high biocompatibility, permeability across the BBB, and high stability *in vivo* (Elsharkasy et al., 2020). Moreover, the surface of EVs can be engineered to increase brain targetability (Marie et al., 2021). Three main types of EVs include apoptotic bodies (ApoBDs), microvesicles (MVs), and exosomes. Major difference among the three types include their formation process, size, and origin (Shao et al., 2018; Nederveen et al., 2021). ApoBDs and most MVs have a large particle size (Elmore, 2007; Shao et al., 2018), making them not ideal candidates for targeted delivery to the CNS. Therefore, authors briefly introduce ApoBDs and MVs, and then concentrate on describing exosomes—the smallest EV type, which may easily pass through the BBB so that it holds a superior potential for CNS-targeted delivery than other EVs (Shao et al., 2018).

Apoptotic bodies are the largest of EVs (500–4,000 nm; Elmore, 2007) that are formed during apoptotic cell disassembly where fragmented cell contents such as organelles and nuclear content are enclosed in membrane-bound vesicles (Santavanond et al., 2021). ApoBDs successfully trigger clearance of damaged cells in tissue development and regeneration of the kidney and bone (Li et al., 2020). MVs are EV subtypes that are released directly from the cell surface of platelets, red blood cells, and endothelial cells (Yang J. et al., 2021) and range in size from 200 to 2,000 nm (Shao et al., 2018). MVs may transport proteins and miRNA between cells and have been used for the diagnosis of autoimmune diseases, treatment of acute kidney injury, etc. (Liu et al., 2016). While ApoBDs and MVs may be tried for CNS-targeted therapy with the help of indirect, BBB penetration-assisting methods, there is limited evidences on the two methods.

Exosomes are the smallest EVs (40–200 nm) that are formed in all types of cells when vesicles bud into endosomes inside cells (Shao et al., 2018). Exosomes may directly penetrate the BBB, are highly stable in peripheral circulation, and thus may protect disease-specific therapeutic molecules, unlike ApoBDs and MVs. At the beginning of researches, exosomes were studied as a biomarker to monitor disease development, allowing early diagnosis and treatment optimization in such stroke, glioma, Parkinson’s diseases, Alzheimer’s diseases, Huntington’s diseases, and amyotrophic scleroses (Liu et al., 2019; Fan et al., 2022). After that period, exosome trials have been extended to therapeutic role for CNS disease as well (Fan et al., 2022). In a Martins et al.’s research, the ability of two Aβ-binding proteins—alpha-1-antichymotrypsin and C4b-binding protein alpha chains—was analyzed to validate exosome levels in patients by using antibody-based methods, indicating significant correlations between alpha-1-antichymotrypsin exosomal concentrations and mini-mental state examination scores and clinical dementia rating scores, indicators of cognitive alterations. Moreover, C4b-binding protein alpha chains was reported to limit the complement activation by Aβ and dead cells in Alzheimer’s disease brains, potentially protecting the neurons from immune responses (Soares Martins et al., 2022). Recently, another notable advantage of exosomes was discovered; their inherited membrane proteins—such as CD9, CD63, prostaglandin F2 receptor negative regulator, and Lamp2b—can be genetically and/or chemically engineered to increase tissue-targetability, showing promising results in preclinical studies for CNS targeting (Sun et al., 2022). In a study by Alvarez-Erviti et al., Lamp2b-RVG plasmids were created to transfect autologous-derived dendritic cells, stimulating the secretion of engineered exosomes. Following that engineering, the exosomes were intravenously injected to mice, resulting in specific delivery of siRNA to neurons, oligodendrocytes, and microglia in the brain, ultimately leading to specific gene (BACE1) knockdown for Alzheimer’s diseases (Alvarez-Erviti et al., 2011). As such, exosomes may be used for targeted delivery of various therapeutics, such as proteins, siRNAs, miRNAs and even drug ingredients of low molecular weight, to injured CNS neurons with the help of genetically and/or chemically engineered surface modification (Sun et al., 2022; Zhou et al., 2022).

Despite such advantages, however, clinically approved CNS-targeted exosomes would require further investigation regarding detailed methods in pointed view of penetration efficiency of the BBB as well as the development of quantitative and qualitative monitoring and imaging tracking methods about targeted delivery to the CNS neurons (Hojun Choi et al., 2022). Moreover, in a research on wound healing promotion using adipose cell-derived exosomes, it was found that the metabolic condition of exosome donors affects the biogenesis, biological activity, and composition of the adipose stem cells (Cianfarani et al., 2013), thus affecting the adipose stem cell exosomes. Technically, isolation and purification methods should also be validated for reliable production in large scale (Rufino-Ramos et al., 2017).

### 2.5. Hybrid nanomedicine

Hybrid NPs are a combination of organic and inorganic NPs, which enable the combined advantages of both types of NPs to be leveraged for the treatment of CNS disease, and these NPs exhibit tunable size and surface charge based on the components included. The size and surface chemistry of NPs are critical since they affect the efficacy of NP delivery to diseased tissues, redistribution of NPs within the body, and potential toxicity (Walkey et al., 2012). One example of the tunability of the size of hybrid NPs is the use of different lipids; in a study comparing antibiotic-loaded lipid-polymer hybrid NPs (LPHNPs) engineered with cationic and zwitterionic lipids, the diameter of cationic LPHNPs was 203 ± 6.6 nm and that of zwitterionic LPHNPs was 191 ± 5.4 nm (Bose et al., 2020). Moreover, when cationic or zwitterionic LPHNPs were introduced into the LPHNP formulation, the charge was reduced by −29 ± 2.1 mV, indicating that LPHNPs are more advantageous in terms of their ability to interact with surrounding cells, their penetration of the BBB, and their colloidal stability (Bose et al., 2020).

High drug-loading yield and sustained drug release are other advantages of hybrid NPs; when the encapsulation efficiencies (%EE) of doxycycline-bare NPs and cationic or zwitterionic lipid layered LPHNPs loaded with doxycycline were compared, the results corresponded to a %EE of 63, 71, and 79%, respectively. Moreover, the antibiotic release rates of cationic LPHNPs at 12, 24, and 48 h were 38, 54, and 66% when zwitterionic LPHNPs were 32, 47, and 61% and doxycycline-bare NPs were 65, 69, and 75%, respectively (Bose et al., 2020).

Hybrid NPs also exhibit high stability and biocompatibility because their outer shells are combinations of the organic and inorganic shells. In an *in vivo* gene delivery study, bare pCas9/methylguanine methyltransferase degraded within 3 min of incubation with DNase I, and pCas9/MGMT plasmids loaded in LPHNPs remained intact throughout varying durations of incubation with DNAse I (Yang Q. et al., 2021). This result indicates that LPHNPs successfully protected pCas9/methylguanine methyltransferase from enzyme degradation and can be used as a stable delivery vehicle (Yang Q. et al., 2021). In addition, the application of LPHNPs after exposure to varying concentrations of cisplatin and curcumin demonstrated a cell viability of >80% based on CCK-9 assay results in A2780 tumor cells (Khan et al., 2020). Nevertheless, for the hybrid NPs, there were only *in vitro* performed studies that depicted *in vitro* or plainly brain cell update; for instance, Fe_3_O_4_ LPHNPs manufactured by self-assembly technique penetrated a BBB model composed of human brain microvascular endothelial cells (BMVECs) under magnetic field guidance (Qiao et al., 2020), and mesoporous silica liposome of poly(acryl acid)-hybrid NPs conjugated with angiopep-2 showed the enhanced penetration rate of the BBB model of BMVECs (Tao et al., 2019). Yet, without *in vivo* studies, it becomes difficult to explain the validity of the targeting experiment.

Despite such advantages, further studies are needed to determine the proper balance of the ratio of the components of the outer shell (i.e., cholesterol). This ratio is important in CNS targeting, as it affects the stability of antigen release, lipid layer stability, and the protection of the integrity of the hybrid structure during long-term storage (Hu et al., 2010). Therefore, recent studies have focused on manufacturing hybrid NPs in an appropriate ligand/receptor density ratio, as this ratio substantially affects their targeting abilities (Alkilany et al., 2019). For example, there was a study in which equimolar amounts of five different lipids—ethyl arachidate, myristic acid, stearic acid, ethyl myristate, and glycerol monostearate—were tested in the form of BBB-crossing terpolymer lipid-hybrid NP, in which polysorbate 80 was covalently attached to poly(methacrylic acid)-grafted-starch, for colloidal stability and *in vitro* application when delivering doxorubicin to glioblastoma multiform (GBM) brain tumor cells. Of the different lipids tested, the ethyl arachidate doxorubicin-hybrid NP showed the most ideal colloidal properties, stability, and highest cytotoxicity against GBM cells (Ahmed et al., 2021). To address the remaining challenges in identifying the ideal out shell composition of hybrid NPs for different CNS therapies, additional studies should focus on determining the proper ratio of the outer shell components in correspondence with degree of *in vivo* stability, targetability, and bioavailability, respectively, in individual types of CNS disease and therapeutics (Table 1).

Similar to the hybrid NPs, hybrid nanomedicine, a combination of NPs and exosomes, has been recently tried, especially in cancer researches; for example, a CD47-expressing exosome cRGD-modified liposome-hybrid nanomedicine inhibited activation of mTOR pathway in ovarian cancer cells indicating targeted codelivery of triptolide and miR_497_ (Li et al., 2022), and another hybrid nanomedicine combining granulocyte-macrophage colony-stimulating factor-overexpressing exosomes and docetaxel-loaded liposomes showed enhanced codelivery of granulocyte-macrophage colony-stimulating factor and docetaxel in metastatic peritoneal cancer rats (Lv et al., 2020). Meanwhile, reported are just few studies showing a potential of hybrid nanomedicine application to CNS-targeted delivery (Rufino-Ramos et al., 2017); for instance, enveloped protein nanocages precisely matching the targeted scaffold via viral glycoprotein were inserted into the EV (Votteler et al., 2016), and exosome-mimetic nanovesicle, in which therapeutic drugs were contained through repetitive macrophage and monocyte breakdown using sequential filtering of extrusion (Jang et al., 2013). However, application of hybrid nanomedicine to CNS-targeted delivery is at its initial stage of development so that it would require further approval in feasibility and validity as the limitations of hybrid NPs do.

In addition to researches on improving the hybrid.nanomedicine themselves, it is expected that future studies on hybrid nanomedicine will be focused on their combination with other indirect methods, such as focused ultrasound (FUS; Ahmed et al., 2021) and photodynamic therapy (PDT; Zhang et al., 2020), approaches of which will hold promise despite the need for additional studies.

## 3. Magnetic field-mediated delivery

This review introduces magnetic delivery in chronological order of their development: magnetic field-mediated passive navigation, magnetotactic bacteria (MTB), magnetic resonance navigation (MRN), and magnetic nanobots. It is important to note that due to preexisting categorical limitations of targeted delivery, this review classify magnetic field-mediated delivery as a direct method, although it actually has indirect characteristics in nature.

Magnetic navigation is a delivery method that accumulates magnetic micro/nanodrug carriers in the targeted area by controlling the direction and propulsion of the carriers with externally provided permanent magnets or electromagnets; this technique has the advantages of navigational delivery to an injury site and exclusive localization within the injury site, allowing therapeutics to escape from emulous binding with non-*in situ* receptors, specifically in the context of CNS-targeted delivery (Chorny et al., 2011). *In vitro* and *in vivo* studies involving centralizing scattered ferromagnetic rods/microcapsules have shown that magnetic forces may be used to successfully manipulate magnetic materials for directional navigation (Nacev et al., 2015; Voronin et al., 2017). Furthermore, magneto-nanobots can be utilized as magnetic field-mediated, actively assisted navigational vehicles that enable cell-specific targeting; in a study where an external magnetic field and magnetic nanobots consisting of magnetic NPs (Fe_3_O_4_NPs) were used to deliver anticancer drugs, the nanobots were shown to gain thrust from O_2_ emissions produced from the decomposition of H_2_O_2_ released from tumor cells (Andhari et al., 2020). Attaching Fe_3_O_4_ to the NPs provides autonomous propulsion and superparamagnetic properties to the nanobot system (Andhari et al., 2020), suggesting a potential of the targeted delivery to deeply located lesions within the CNS. However, using an external magnetic field is difficult in tissues greater than 2 cm deep within the body, as the aforementioned advantages of magnetic field-mediated directional navigation sharply decrease with increasing distance between the magnets and the carriers (Hwang, 2020). Although internal methods such as the implantation of magnets within the body have been devised as an alternative (e.g., intrathecal implant; Lueshen et al., 2015), implants can trigger side effects such as infection and intolerance to them (Ganz, 2017). Thus, these disadvantages of external and internal methods should be augmented to promise better magnetic field-mediated delivery.

Magnetotactic bacteria are groups of bacteria that contain magnetosomes, which are intracellular structures that consist of biological materials such as magnetite (Fe_3_O_4_) and greigite (Fe_3_S_4_); MTB-derived magnetosomes thus have higher biocompatibility than artificial NPs due to their biological properties (Alphandery et al., 2017). Magnetosomes can be isolated from MTB and used as drug delivery carriers, and, as an alternative to paramagnetic/superparamagnetic NPs, MTB themselves can be loaded with drug-carrying vehicles for magnetic field-mediated navigation into target tissues. In one recent study, when magnetosome toxicity was evaluated in mice, complete blood counts and the results of a basic metabolic panel were within the normal range, and no change in the composition of urine or weight was found (Nan et al., 2021). Additionally, drug-carrying MTB with long actin-like filaments encoded by the mamK gene exhibit magneto-aerotaxis, which enables them to align with the direction of an applied magnetic field, move toward the hypoxic regions of targeted cells, and directly navigate the MTB-loaded vehicles to the CNS (Marcuello et al., 2018). In addition, magnetosomes have been shown to promote the axonal outgrowth of neurons through stretch growing when magnetic fields are applied (De Vincentiis et al., 2021). In one study, a total of 500–700 μg of magnetosomes coated with poly-L-lysine and magnetic fields of 202 kHz and 27 mT were used to treat GBM brain tumor cell models, and living GBM cells in all mice completely disappeared after 68 days (Alphandery et al., 2017). Although MTB have many advantages, they may adversely affect the human immune system by decreasing the level of lymphocyte proliferation (Nan et al., 2021). The composition of magnetosomes produced in MTB is often difficult to reproduce, leading to their low production yield (Alphandery et al., 2017). To overcome these limitations, MRN was developed.

Magnetic resonance navigation is a delivery method that uses a magnetic resonance imaging (MRI) scanner to provide propulsion to microcarriers (up to 400 mT/m) and to make them follow a preprogrammed route to the targeted area (Martel, 2010). Administrating MRN consists of three steps—steering, creating a magnetic gradient, and real-time tracking imaging—that are repeated until the carrier reaches the target (Pouponneau et al., 2014; Li et al., 2019). In a study testing MRN targeting in a two-level bifurcation, magnetic drug-eluting beads aggregated with targeting success rates of 84, 100, 84, and 92% for the four different ends of the branch routes (Li et al., 2019). In another study on rabbit hepatic arteries, the MRN successfully steered therapeutic magnetic microcarriers to the left lobes (Pouponneau et al., 2014). In these studies, MRN showed targeting effectivity both in the one-level bifurcation in an animal model and in the higher level of bifurcation of artificial pathways; these findings demonstrate its potential use in targeting more complex pathways in the human body (Pouponneau et al., 2014; Li et al., 2019). However, the references currently cited are based on the treatment of hepatocellular carcinoma by liver chemoembolization. Hence, it is unsure how will it perform well for the brain, which should be evaluated in future studies.

Furthermore, factors such as pulsatile flow can currently limit the use of MRN in *in vivo* studies to even simple pathways, such as one or two Y-bifurcations and 2D vascular phantoms (Folio and Ferreira, 2017). Moreover, some nonspherical microparticles navigated using MRN occasionally attach to the surface of nontarget tissues (Ghosh et al., 2021). However, this problem may be solved by increasing the magnetic gradient to overcome friction and by administering orthogonal pulses (Ghosh et al., 2021). While limitations exist, these studies demonstrate the targeting effectivity and potential of MRN for use in more complex pathways, making it one of the most promising tools for CNS-targeted therapy (Table 2). However, it should be in mind that it comes from the authors’ personal extrapolation from the current information and their use in clinical paradigms cannot be justified yet due to such major limitations.

**Table 2:** Magnetic field-mediated delivery.

Type	Advantage/Disadvantage		Authors	Subject	Result
Magnetic field-mediated passive navigation using external magnetic field	Advantages	Magneto-nanobots allows cell-specific targeting	Andhari et al. (2020)	External magnetic field and magnetic nanobots consisting of Fe3O4 NPs	Provides autonomous propulsion ability and superparamagnetic property to the nanobot system
	Disadvantages	Sharply reduced effective delivery as the distance between the magnets and the carriers increases	Hwang (2020)		Unable to target tissues greater than 2 cm deep within the body
		Internal methods caused unexpected side effects to the body	Ganz (2017)	Magnetic implants around the gastroesophageal junction	Caused dysphagia, persistent nausea, and postoperative nausea
MTBs	Advantages	Higher biocompatibility than artificially synthesized NPs	Alphandery et al. (2017)	Magnetosomes consist of biological materials such as magnetite (Fe3O4) and greigite (Fe3S4) crystals	
		Less toxicity	Nan et al. (2021)	Tested its toxicity with mice	Complete blood count and basic metabolic panel showed normal range, and no change in the composition of urine and weight was found
		Exhibit magneto-aerotaxis which allows the drug to be directly navigated to the brain across the BBB when conjugated with NPs	Alphandery et al. (2017)	Magnetosome coated with poly-L-lysine (M-PLL) that contains 500–700 μg of iron was administered in a magnetic field of 202 kHz and 27 mT to treat GBM	Living GBM cells completely disappeared after 68 days for all treated mice
	Disadvantages	May adversely affect the human immune system	Nan et al. (2021)	Regarding the decreased level of lymphocyte proliferation	
		Difficulty in reproducing the composition of magnetosome and its low production yield	Alphandery et al. (2017)		
MRN	Advantages	Targeting effectivity both in the one-level bifurcation of an animal model and in the higher level of bifurcation of artificial pathways	Li et al. (2019)	Testing the MRN in a two-level bifurcation phantom with magnetic drug-eluting beads aggregates	Showed targeting success rates of 84, 100, 84, and 92%
			Pouponneau et al. (2014)	Rabbit’s hepatic artery to the left/right liver lobes	Successfully steered therapeutic magnetic microcarriers to the left lobes
	Disadvantages	As pulsatile flow currently limits MRN to simple pathways *in vivo* studies	Folio and Ferreira (2017)	Applied to one or two simple Y-bifurcations or only 2D vascular phantoms	
		Some non-spherical microparticles occasionally attach to the surface of tissues and cannot travel to the target	Ghosh et al. (2021)		

CNS, central nerve system; BBB, blood–brain barrier; MTBs, magnetotactic bacteria; GBM, glioblastoma; and MRN, magnetic resonance navigation.

## 4. Indirect targeted delivery

The most considerable barrier to the use of direct targeted delivery has been their inability to allow therapeutics that exceed 400 Da to pass through the BBB (Pardridge, 2012; Cui and Cho, 2022). To overcome this disadvantage of the direct targeted delivery, development of indirect targeted methods has been demanded. In this section, the authors aim to introduce indirect chemical and physical methods that open the BBB, the mechanisms of each method, their advantages and disadvantages, and discussion of the recent findings with important implications. Chemical and physical methods can increase the permeability of the BBB using chemicals and physical energy, respectively. Mannitol has been widely used to decrease intracranial pressure (Chengyan Chu et al., 2018), but shown side effects such as edema and renal failure (Visweswaran et al., 1997) and a short efficacy time (Cosolo et al., 1989). Therefore, other chemicals, such as bradykinin and 1-O-pentylglycerol, have been studied. After briefly introducing chemical methods, this section mainly focuses on describing two main physical methods that are being developed: FUS and LASER therapy.

### 4.1. Chemical delivery

Indirect chemical targeted delivery is a method that chemically increases the permeability of the BBB, thereby allowing therapeutics to pass the BBB and take effect in the CNS (Pardridge, 2012; Dong, 2018; Cui and Cho, 2022). Hyperosmotic mannitol is the most widely used BBB permeabilizer (Chengyan Chu et al., 2018). It triggers dehydration of BBB endothelial tissues and deflates cell bodies, causing loosening of tight junctions, which enhances the permeability of drugs, stem cells, liposomal vehicles, and antibodies (Hendricks et al., 2015). However, when mannitol was injected into adult Sprague–Dawley rats at the optimum rate of 0.25 ml.s-1.kg-1 for 20 s, BBB disruption has been shown to last for approximately 5 min at maximum and then rapidly reverse (Cosolo et al., 1989), and administering 1,171 ± 376 g mannitol to a patient with normal baseline renal function resulted in acute renal failure (Dorman et al., 1990). Moreover, intracarotid arterial hyperosmolar mannitol increased the production of cytokines, chemokines, and trophic factors, leading to a neuroinflammatory response (Burks et al., 2021). Therefore, as mannitol has a short efficacy time and potential provocation of renal failure and systemic toxicity, and causes a neuroinflammatory response, other chemical methods are being developed to overcome such limitations.

Bradykinin is another agent used to permeabilize the BBB. Bradykinin is a vasoactive compound that selectively increases the permeability of abnormal brain capillaries (Black, 1995). A study using an RG2 rat glioma model demonstrated that stimulating the B2 bradykinin receptor rapidly increases the permeability of the BBB, especially in brain-tumor-associated tissue (Bartus et al., 1996). Moreover, treating female Wistar rats with bradykinin caused a 3.3-fold increase in the number of small arterioles, a 2.1-fold increase in the number of medium arterioles and a 1.5-fold increase in the number of large arterioles, alluding to an increase in pinocytotic vesicle density and BBB permeability (Raymond et al., 1986). However, it has a short half-life (27 ± 10 s; Cyr et al., 2001), may decrease the blood flow to the cerebrum, and causes extravasation, necrosis, edema, and BBB breakdown at high dosages or pathological conditions, since it is a potent vasoactive compound for arterial dilation (Wahl et al., 1999). To overcome such limitations, bradykinin receptor agonist NG291 is being developed to induce a rapid onset of transient BBB disruption without early brain injury. In a Sprague Dawley rat and CD-1 mouse model study, notable NG291-induced increase in BBB permeability was revealed in a localized, reversible, dose-dependent and P-gp efflux transport-mediated manner. Moreover, NG291 did not evoke short-term neurotoxicity, nor the increase of brain water content, the number of Fluoro-Jade C positive cells, and astrocyte activation (Rodríguez-Massó et al., 2021), indicating that biased agonism may play a role in enabling therapeutics to specifically target BBB opening without causing brain injury.

1-O-pentylglycerol is an alkylglycerol that has also been investigated for its ability to permeabilize the BBB. 1-O-pentylglycerol affects the endothelial cell morphology of the barrier and disrupts the BBB structure (Hülper et al., 2013). For example, an *in vivo* tumor-free and C6 glioma-bearing rat experiment indicated that intracarotid injection of 1-O-pentylglycerol caused a transfer of fluorescein and lissamine-rhodamine B200 across the BBB, as the mean ratio of ipsilateral to contralateral fluorescence intensity in the coronal sections for fluorescein was 6.45 ± 1.4, and the mean ratio of lissamine-rhodamine B200–albumin was 2.66 ± 1.0 (Erdlenbruch et al., 2003), which indicates applicability as one of brain tumor therapeutics. Additionally, no 1-O-pentylglycerol accumulation was found in the brain, and more than 70% of the administered dose was excreted within 270 min after administration, which suggests rapid renal elimination and the nontoxicity of 1-O-pentylglycerol (Erdlenbruch et al., 2005). While 1-O-pentylglycerol raised concerns regarding immunogenicity and thus adverse effects in nontarget cells, incubating bEnd3 mouse brain cells with poly(alkyl cyanoacrylate)-alkylglyceryl dextran did not show significant toxicity at concentrations <25 μg/mL (Ibegbu et al., 2017).

Although various indirect chemical targeted delivery methods exist and are under development, it is not easy to reach a consensus regarding a specific chemical to recommend. The most researched and used chemicals—mannitol, bradykinin, and 1-O-pentylglycerol—each has unignorable shortcomings, such as short efficacy time (Erdlenbruch et al., 2005), drug leakage (Rodríguez-Massó et al., 2021), and systemic toxicity (Dorman et al., 1990). Due to the aforementioned significant side effects, their use in clinical paradigms cannot be justified yet because of such major limitations. Therefore, indirect physical targeted delivery methods are being developed to remedy such limitations.

### 4.2. Physical delivery

#### 4.2.1. Focused ultrasound

Focused ultrasound is a noninvasive augmentation method that opens the BBB through the application of high-intensity focused ultrasound (HIFU), which helps deliver therapeutics to the targeted brain region. When the FUS wave is applied in a pulsed manner, tissues experience a cycle of high and low pressure (Timbie et al., 2015). FUS is used to generate microbubbles, which act as contrast media, and these bubbles function as echo-enhancers that pass through the ultrasound field, resulting in stable cavitation (Lee et al., 2017). Then, the blood vessels are mechanically stimulated by the microbubbles, which leads to opening of the BBB. Since the microbubbles can concentrate the ultrasonic energy, the initial ultrasound pressure needed for opening the BBB is considerably reduced, leading to the BBB opening with lesser risks of skull bone heating (Burgess et al., 2015).

Focused ultrasound increases the bioavailability of therapeutics in the target area and helps them maintain their bioactivity throughout the delivery processes (Huttunen and Saarma, 2019). In a recent mouse study of Parkinson’s disease, FUS was used with two sonication locations in regions that showed severe damage in Parkinson’s disease: the caudate-putamen and substantia nigra (Dauer and Przedborski, 2003), and varied pulse lengths of 5,500 cycles (3.3 ms) to 10,000 cycles (6.6 ms) were used. When neurturin, a type of neurotrophic factors, was directly injected, its bioavailability area was limited to an average of 0.20 ± 0.05 mm^2^. However, with FUS, the bioavailability of neurturin was 5.07 ± 0.64 mm^2^ in the caudate-putamen and 2.25 ± 1.14 mm^2^ in the substantia nigra, showing a maximum 25-fold increase due to BBB opening, allowing neurotrophic factors to pass through (Samiotaki et al., 2015). Additionally, FUS causes minimal damage to the nervous system while increasing the delivery rate of therapeutics passing through the BBB. In a study of nonhuman primates in which the short-term (2 days) and long-term (18 days) effects on FUS-mediated BBB opening and the secondary effect on visual-motor cognitive task-related touch accuracy or reaction time were assessed, response accuracy did not decrease in the short term (from 0.76 ± 0.08 to 0.80 ± 0.03) and nor in the long term (from 0.62 ± 0.08 to 0.68 ± 0.05), which suggests that cognitive performance dose not worsen following the FUS-induced BBB opening in nonhuman primates. In addition, no observable damage, microhemorrhage, or necrosis was observed in the histological test after the nonhuman primates were euthanized (Pouliopoulos et al., 2021). The aforementioned results suggest that FUS should be evaluated in healthy human populations to check whether it causes deterioration of cognitive functions or additional damage to the nervous system.

Although FUS has advantages, there are also limitations in applying FUS in clinical practices, especially regarding intensity. HIFU treatment with a frequency of 20 MHz generating a focal intensity ranging of 1,000–10,000 W/cm^2^ may induce skin burns and neuronal damage due to thermal injuries caused by the inverse piezoelectric effect, leading to irreversible coagulative necrosis (Fan et al., 2012). In contrast to HIFU, low-intensity focused ultrasound (LIFU) with a peak intensity ranging of 10–500 W/cm^2^ can increase the penetration of the therapeutics but may not cause side effects such as microhemorrhage and ischemia (Meng et al., 2018). Regarding the safety margin of FUS, LIFU treatment with a frequency ranging of 220–650 KHz producing a focal intensity of 80 W/cm^2^ through an intact cranium induced no significant hemorrhage or necrosis in the necropsic cortex of eight 3–4-month-old swine (Zibly et al., 2014). Therefore, controlling the intensity and establishing clinical evidence is necessary for FUS to be an effective treatment. In authors’ personal opinion, the focal peak intensity ranging of 10–80 W/cm^2^ is ideal for FUS-based indirect delivery of therapeutics into the CNS in term of safety and efficiency: the stronger intensity, the better permeability but more adverse effects. Nevertheless, if more animal studies can be performed and the results applied to humans, FUS may be an effective way to help deliver therapeutics in a minimally invasive manner.

#### 4.2.2. Light amplification by the stimulated emission of radiation therapy

Light amplification by the stimulated emission of radiation (LASER) therapy has been used in neurosurgery to destroy unhealthy brain tissue by creating heat. However, excess heat can induce damage to neighboring nontarget tissues. Therefore, various studies have been conducted to increase the spatial precision of LASER therapy, leading to the development of CNS-targeted therapy that includes using LASER to change cells into a specific state in which LASER increases the permeability of the BBB and subsequently deliver therapeutics. Currently, LASER interstitial therapy (LITT), PDT, and photobiomodulation therapy (PBM) are three approaches to LASER therapy that are studied for CNS-targeted delivery. LITT is a technique used to increase local BBB permeability by directing a LASER to the target area, transmitting light energy through a thin fiber, which is then converted into thermal energy, and creating a local disruption of the BBB (Melnick et al., 2021). LITT has been applied to the treatment of CNS diseases such as GBM (Patel et al., 2020). LITT-induced hyperthermia disrupts the BBB, with peak permeability for anti-neoplastic drugs happening by 1–2 weeks following ablation and disappearing by 4–6 weeks (Leuthardt et al., 2016). However, there are difficulties in controlling temperature to prevent thermal damage to nearby organs or structures (Salem et al., 2019); specifically, high temperature—close to or above 100°C—at the tissue can cause water to boil, and undesirable air bubbles can be produced (Zhu et al., 2017). To overcome this problem, thermal MRI has been introduced to monitor the heating process in real time and deactivate the system when the temperature reaches a critical level (Salem et al., 2019; Chen et al., 2021). However, well-designed prospective clinical trials and appropriate follow-up time are required to check patient outcomes with using LITT.

Another type of LASER therapy is PDT, which consists of three major elements: photosensitizers, light energy, and tissue oxygen. When a specific wavelength of light activates the photosensitive agent, the photosensitizer generates cytotoxic reactive oxygen species (ROS) such as hydroxyl radicals and singlet oxygen. With the help of photosensitizers such as 5-aminolevulinic acid and phthalocyanines, PDT can site-selectively open the BBB by destroying tight junction proteins and increasing the tight junction gap (Semyachkina-Glushkovskaya et al., 2020). However, although PDT can increase BBB permeability, it cannot utilize near-infrared light, making it difficult to allow deep penetration and low phototoxicity to normal tissues (Lan et al., 2019). Therefore, two-photon excitation and upconversion NPs, such as angiopep-2 conjugated NPs, are being studied to minimize invasiveness (Tsai et al., 2018).

The mechanism of PBM action in cells is the same as that observed in the use of PDT, but the two have apparent differences. PDT requires exogenous photosensitizers, and its high level of ROS generation causes cellular damage, while PBM utilizes endogenous cellular photosensitizers, resulting in low ROS levels that may not deteriorate cellular functions (Lubart and Friedmann, 2011). Furthermore, the combination of PDT and PBM can act as a synergistic therapeutic tool with the help of increased efficacy of anti-neoplastic treatment by increasing ATP production in mitochondria and increasing the production and homogeneity of protoporphyrin X, which is an endogenous photosensitizer required in PDT (Joniová et al., 2021). Additionally, in a study, PDT (660 nm) combined with PBM (850 nm) in which the Ru complex was used as a photosensitizer, increased photocytotoxicity was observed in A375 tumor cells (Negri et al., 2019). However, in the transcranial PBM approach, light cannot penetrates the brain more than 20mm from the cortex, making it difficult to use in delivering sufficient doses to the targeted region (Moro et al., 2014). Further study and clinical tests are required to fully understand the mechanism of PBM, solve these limitations, and improve the efficacy of PBM.

## 5. Limitations/challenges

Direct and indirect targeted deliveries have many advantages and a great potential for use in CNS disease treatments, but have some limitations to be overwhelmed. Organic NPs require complicated process in production, and accumulate in the liver and spleen, and have a short half-life, and inorganic NPs have systemic toxicity involving multiple organs to be solved. Exosomes need further studies to figure the dependency of their characteristics on progenitor cells and nonstandardization of isolation and purification out. Hybrid nanomedicine demands the appropriately balanced ratio of the outer shell components, such as ligand/receptor density ratio, for the stability. Also, magnetic field-mediated delivery has difficulty in maintaining magnetic field greater than 2 cm deep within the body and navigational ability properly with increasing distance, MTB may trigger activation of the human immune system, and MRN has the obligation to use pulsatile flow, which disturbs its constant magnetic guidance even in simple pathways, and the tendency of nonspherical microparticles to attach to nontarget tissues surfaces. Regarding challenges of indirect methods, chemical deliveries, such as bradykinin and 1-O pentylglyceol, have numerous disadvantages; a short half-life, extravasation, adverse effects in nontarget organs. FUS has safety issues, intensity-related thermal injury by the inverse piezoelectric effect, and LASER therapy has problems of phototoxicity and generation of local hyperthermia and ROS to be solved.

Meanwhile, many of the delivery methods, especially NPs, described in the current manuscript have been known to reduce libido; for example, silver NPs compromised reproductive activity such as sperm motility 7 days after intravascular injection in rabbits (Castellini et al., 2014). Similarly, treatment of unconjugated titanium, gold, and silver NPs reduced the motility and viability of cryopreserved bovine spermatozoa from post-equilibration to post-thaw period following ejaculation (Pande et al., 2022). Furthermore, gender difference was reported in hepatic toxicity, especially dominant in female sex, after oral exposure of titanium NPs over 90 days in rats (Chen et al., 2019). Also, male sex-dominant differences in histopathology and mortality were noticed after single oral administration of copper NPs at higher dose in rats (Lee et al., 2016). To the contrary, no gender difference was revealed in bone marrow toxicity after oral exposure of silver NPs for 28 days in rats (Kim et al., 2008). Now, there are few clinical studies investigating gender difference of NPs; interestingly, in a randomized controlled clinical trial in which 19 patients with carious teeth were recruited, subgroup analysis showed no gender difference in marginal integrity restoration of resin composites following pretreatment with silver and gold NPs (Nemt-Allah et al., 2021). Hence, it requires further studies prior to discussion about their gender based effect.

## 6. Future directions and conclusion

In direct CNS-targeted delivery, organic NPs show high biocompatibility and low immunogenicity but are difficult to produce at a large scale, lack stability, and are eliminated too early. Inorganic NPs show a high drug-carrying capacity, surface modifiability, and magnetic field-mediated navigability, but have toxic side effects. To overcome these limitations, efforts have been made to control the components of outer shells for better stability and reduce factors that cause toxicity. Although hybrid NPs, a combination of organic and inorganic NPs, have many advantages, such as tunable size, high stability, and high drug-loading yield, they have side effects, including harmful tissue deposition patterns, unintended activation of the host immune response, and damage to nontarget cells. Among EVs, exosomes show a notable potential for CNS-targeted delivery of therapeutics because of genetically and/or chemically engineered targetability of inherited surface proteins as well as biocompatibility and permeability across the BBB. Therefore, studies are in progress to manufacture NPs, exosomes, and ligands in an appropriate ratio, and contingencies such as PEG stealth technology have evolved, making hybrid nanomedicine, a combination of NPs and EVs, an effective direct delivery method. Another advantageous direct delivery method is magnetic field-mediated delivery, which utilizes magneto-nanobots, MTB, and MRN. However, methods using an external magnetic field have difficulty reaching deep targeted regions, and MTB negatively affect immune system of the human body and have a low production yield. Therefore, MRN was developed to overcome these issues, showing successful targeting efficacy in simple pathways in *in vivo* studies. While further studies are needed to apply MRN in more complex pathways, due to its pulsatile flow-induced navigability weakening in real time, it is one of the most promising direct CNS-targeted delivery methods.

**Figure 2:**
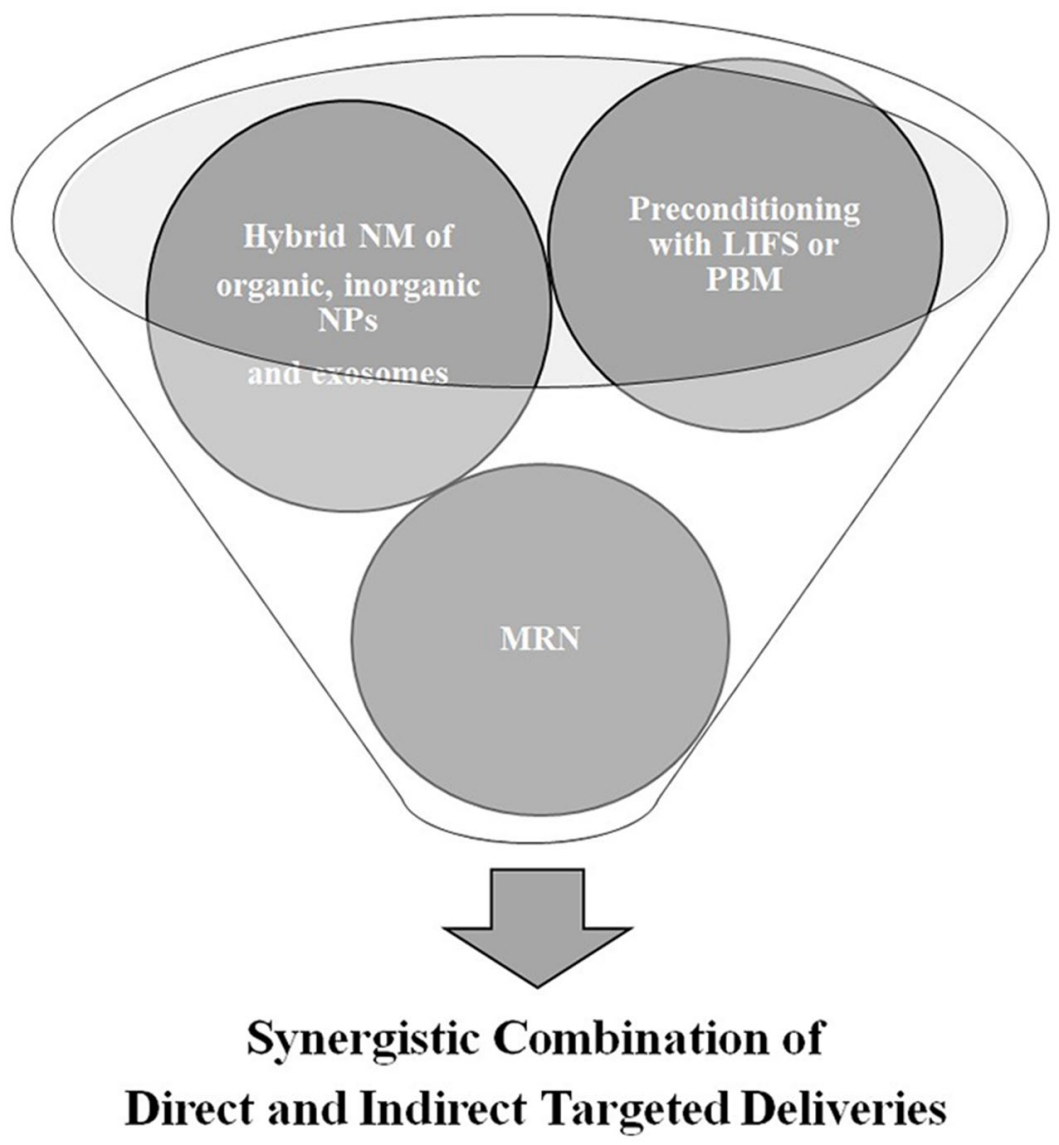
Future directions of targeted delivery of therapeutics in central nervous system diseases; low intensity focused ultrasound (LIFS); photobiomodulation therapy (PBM); nanomedicine (NM); nanoparticles (NPs); and magnetic resonance navigation (MRN).

As there are obstacles to overcome in the use of direct CNS-targeted delivery methods, indirect delivery methods have been developed and are used together with direct methods to create a synergistic effect. Chemicals such as mannitol and bradykinin effectively increase the BBB permeability but have a short half-life and show toxicity when injected. Although short-chain alkylglycerols do not have the same drawbacks as mannitol and bradykinin, they lead to complications related to immunogenicity problems and can invade nontarget cells. While FUS, one of the physical targeted delivery methods, increases and maintains the bioavailability of therapeutics and causes minimal damage to the CNS, it has problems related to delivery intensity that need to be solved to make this method more advantageous. LASER therapies, such as LITT, PDT, and PBM, have recently gained prominence and have been increasingly used for disrupting the BBB. However, they still have limitations—thermal damage, shallow penetration, and phototoxicity—to overcome, so thermal MRI is being introduced in LASER therapies, and two-photon excitation and upconversion NPs are being studied. Based on the outcomes, such as anti-neoplastic activity and validity of endogenous photosensitizers, LASER therapy may be one of the most promising indirect CNS-targeted therapy methods. While this article has recommended the most promising direct and indirect targeted therapies, it is important to note that combining the direct and indirect methods, such as hybrid nanomedicine of organic, inorganic NPs, and exosomes via MRN following preconditioning treatment with either PBM or LIFS, will be more effective and focused in the future (Figure 2).

## Author contributions

JA, SW, HS, SI, GY, SL, K-iK, and CH substantially contributed to all of the following aspects of this study: (1) study conception and design, acquisition of data, or analysis and interpretation of data, (2) drafting or critical revision of the article for important intellectual content, (3) final approval of the version to be published, and (4) agreement to be accountable for all aspects of the work in ensuring that questions related to the accuracy or integrity of any part of the work are appropriately investigated and resolved. All authors contributed to the article and approved the submitted version.

## Funding

This work was supported by the 2022 Research Fund of University of Ulsan, Ulsan, Republic of Korea. The study sponsor had no involvement in the study design; in the collection, analysis, or interpretation of data; in the writing of the manuscript; or in the decision to submit the manuscript for publication.

## Conflict of interest

The authors declare that the research was conducted in the absence of any commercial or financial relationships that could be construed as a potential conflict of interest.

## Publisher’s note

All claims expressed in this article are solely those of the authors and do not necessarily represent those of their affiliated organizations, or those of the publisher, the editors and the reviewers. Any product that may be evaluated in this article, or claim that may be made by its manufacturer, is not guaranteed or endorsed by the publisher.
